# Venous Congestion in Transmesenteric Hernia After Superior Mesenteric Artery Stenting for Chronic Mesenteric Ischemia

**DOI:** 10.7759/cureus.84743

**Published:** 2025-05-24

**Authors:** Vishnu Prasad Pulappadi, Santhosh Poyyamoli, Kembai Shanmugam Rajkumar, Pankaj Mehta, Mathew Cherian

**Affiliations:** 1 Interventional Radiology, Kovai Medical Center and Hospital, Coimbatore, IND; 2 General Surgery, Kovai Medical Center and Hospital, Coimbatore, IND

**Keywords:** chronic mesenteric ischemia, internal hernia, reperfusion, stent, superior mesenteric artery

## Abstract

Endovascular stenting is commonly done for chronic mesenteric ischemia (CMI). We report a case of venous congestion in transmesenteric hernia following superior mesenteric artery (SMA) stenting for CMI. A 52-year-old man with a history of extended right hemicolectomy presented with post-prandial abdominal pain for two months. Computed tomography (CT) revealed celiac axis occlusion, high-grade ostial stenosis of the superior and inferior mesenteric arteries, and transmesenteric hernia with volvulus. The patient underwent SMA stenting, and his post-prandial pain resolved. Two days later, he developed right iliac fossa pain. CT revealed the dilatation of mesenteric veins, lymphadenopathy, and fat stranding within the hernia sac, and ascites, suggesting venous congestion and impending strangulation. The patient underwent laparotomy and hernia repair, following which his symptoms resolved.

## Introduction

Chronic mesenteric ischemia (CMI) is characterized by post-prandial abdominal pain, fear of eating, and weight loss. It is more common in the elderly, and the most common cause is atherosclerosis. As surgical bypass is associated with a high complication rate and morbidity, stenting has become the preferred treatment option for CMI [1].

Reperfusion injury is a complication that occurs after the reperfusion of the ischemic bowel. It occurs due to the generation of reactive oxygen species, neutrophil activation, and release of inflammatory cytokines [2]. Identification of bowel infarction is essential before mesenteric revascularization to prevent complications related to reperfusion injury. Although it occurs in up to 44% of cases after reperfusion in acute mesenteric ischemia [3], it is rare after mesenteric artery stenting for CMI [4-8]. The imaging features of reperfusion injury are bowel wall thickening, mucosal hyperenhancement, submucosal edema, mesenteric fat stranding, and inter-bowel free fluid [3]. Treatment of reperfusion injury is challenging, and resection of infarcted bowel may be necessary if clinical deterioration occurs despite supportive care, which includes fluid resuscitation, bowel rest, and antibiotics. 

A transmesenteric hernia is an internal hernia in which small intestinal loops herniate through a congenital or surgically created mesenteric defect. While it develops most commonly after Roux-en-Y anastomosis, it can also develop after ileocolic anastomosis [9,10]. Strangulation is more common in transmesenteric hernias than other internal hernias [9].

The development of venous congestion within an internal hernia after mesenteric revascularization has not been reported previously. We report a case of venous congestion in transmesenteric hernia following superior mesenteric artery (SMA) stenting for CMI.

## Case presentation

**Figure 1:**
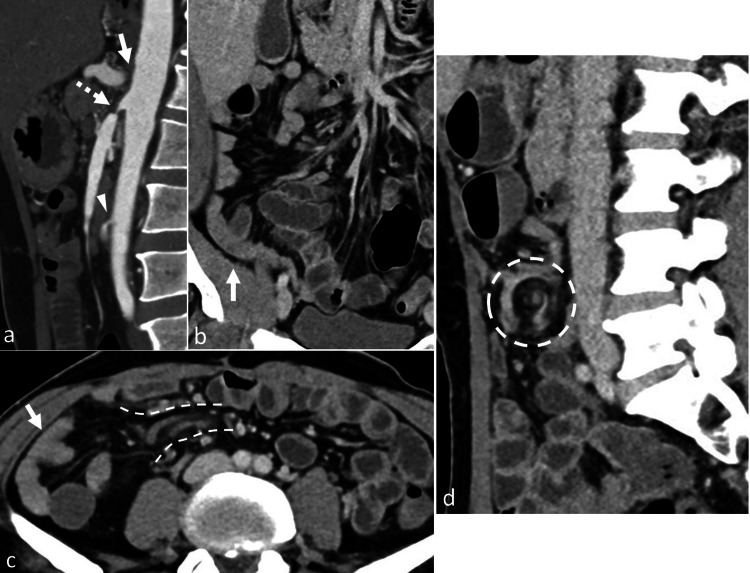
Chronic mesenteric ischemia with transmesenteric hernia (a) Sagittal reformatted arterial phase CT image showing the short-segment occlusion of the celiac axis ostium (arrow) and short-segment high-grade stenosis of the ostia of the superior (dashed arrow) and inferior (arrowhead) mesenteric arteries. (b, c) Coronal reformatted (b) and axial (c) venous phase CT images showing the clustering of ileal loops in the right iliac fossa (arrows), with a perpendicular orientation of its vascular pedicle (outlined by dashed lines in c) to the other branches of the superior mesenteric artery, suggestive of transmesenteric hernia. (d) Sagittal reformatted venous phase CT image showing the swirling of the vascular pedicle (outlined by dashed circle) of the hernia sac CT: computed tomography

A 52-year-old man presented with the complaint of post-prandial abdominal pain for two months. He had undergone an extended right hemicolectomy one year ago for transverse colon perforation secondary to cytomegalovirus colitis. He was a chronic smoker with no other comorbidities. Evaluation done for immunocompromised states was negative. On examination, his abdomen was soft and non-tender with no guarding or rigidity. Multiphase contrast-enhanced computed tomography (CT) revealed the short-segment occlusion of the celiac axis and high-grade ostial stenosis of the superior and inferior mesenteric arteries (Figure 1a). CT also revealed the clustering of ileal loops in the right iliac fossa (Figure 1b, 1c), with a perpendicular orientation of its vascular pedicle in relation to the other branches of SMA on axial images, suggesting an internal hernia through the mesenteric defect created during the prior surgery. There was swirling of the vascular pedicle on sagittal images (Figure 1d), suggesting volvulus. However, the clustered ileal loops showed normal enhancement. Because his symptoms were attributable to CMI rather than internal hernia, the patient was planned for SMA stenting.

**Figure 2:**
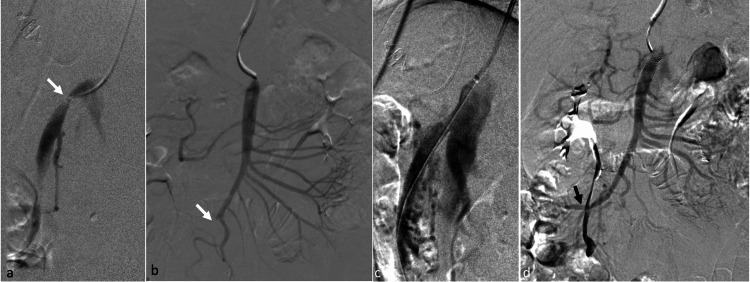
SMA stenting (a) Lateral SMA angiogram showing high-grade stenosis near its ostium (arrow). (b) Antero-posterior SMA angiogram performed after crossing the stenosis showing occlusion in the distal SMA (arrow). (c) Lateral SMA angiogram after stent placement showing no significant residual stenosis in the proximal SMA. (d) Antero-posterior SMA angiogram after stenting showing the recanalization of the distal SMA and its distal ileal and ileocolic branches (arrow) SMA: superior mesenteric artery

The patient was given a loading dose of 300 mg of aspirin and 300 mg of clopidogrel the day before the stenting. An angiography performed through right brachial artery access confirmed high-grade stenosis in the SMA (Figure 2a). After crossing the stenosis, an angiogram was done, which showed a tapered occlusion of the distal segment of the SMA, likely at the neck of the transmesenteric hernia (Figure 2b). Following pre-dilatation using a 4×40 mm balloon (Advance 35LP, Cook Medical, Bloomington, IN, USA), a 9×27 mm balloon-expandable stent (Express LD, Boston Scientific, Marlborough, MA, USA) was placed across the site of stenosis (Figure 2c). The distal segment of the initially occluded SMA and the ileocolic and distal ileal branches had opened up following stenting (Figure 2d). After the procedure, the patient was kept on 150 mg of aspirin and 150 mg of clopidogrel once daily.

**Figure 3:**
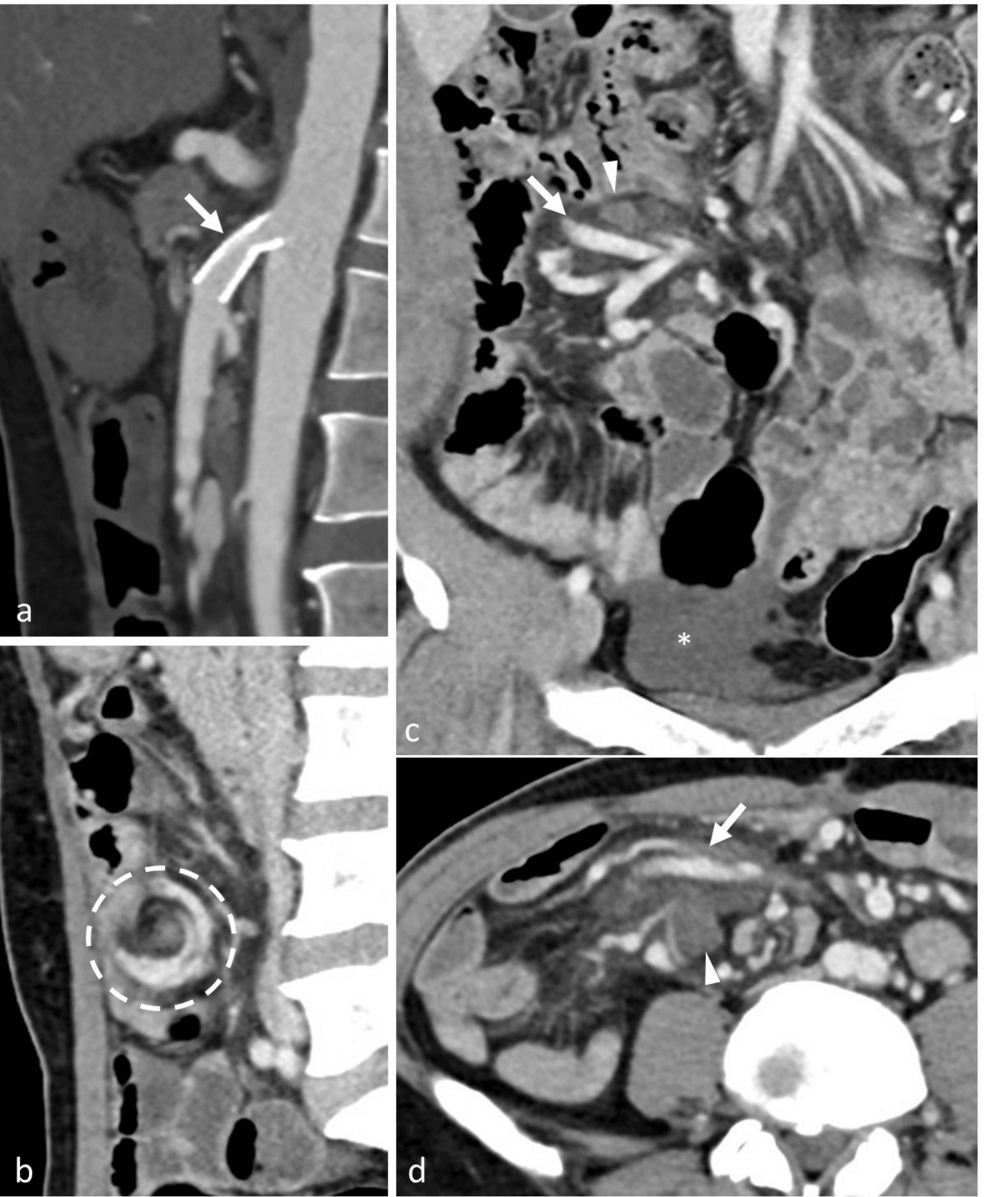
Venous congestion in the hernia sac after SMA stenting (a) Sagittal reformatted arterial phase CT image showing patent SMA stent (arrow). (b) Sagittal reformatted venous phase CT image showing persistent volvulus of the hernia sac (outlined by dashed circle). (c, d) Coronal reformatted (c) and axial (d) venous phase CT images showing engorgement of the mesenteric veins (arrows), lymphadenopathy (arrowheads), and fat stranding in the hernia sac along with mild ascites in the pelvis (asterisk in c) SMA: superior mesenteric artery; CT: computed tomography

Although the patient's post-prandial pain resolved after the stenting, two days later, he developed pain in the right iliac fossa, gradually increasing in severity and unrelated to food intake. The differential diagnoses considered at this stage were stent thrombosis and reperfusion injury. Contrast-enhanced CT revealed a patent SMA stent (Figure 3a) and persistent volvulus of the hernia sac (Figure 3b). There were new-onset dilatation of mesenteric veins, lymphadenopathy, and fat stranding within the hernia sac, and mild ascites, suggesting venous congestion and impending strangulation (Figure 3c, 3d). The increased inflow of arterial blood after the SMA stenting would have resulted in venous congestion. All bowel loops showed normal wall enhancement. The patient was planned for laparotomy as the abdominal pain was persistent. The antiplatelet agents were stopped three days before the surgery, and intravenous heparin infusion was started at the rate of 1000 units per hour, with the monitoring of activated partial thromboplastin time, to prevent stent thrombosis. Heparin infusion was stopped six hours before the surgery. The diagnosis of transmesenteric hernia with volvulus was confirmed intra-operatively. Excision of the hernia sac, hernia reduction, and mesenteric defect repair were done. No bowel resection was done as the ileal loops in the hernia sac were viable. The patient had complete resolution of symptoms following the surgery. Dual antiplatelet therapy was restarted on the next day after the surgery. During the follow-up visit after one month, the patient had no recurrent symptoms.

## Discussion

We observed a rare complication of venous congestion in an internal hernia after SMA stenting. Our patient had an internal hernia through the mesenteric defect that was created during a previous surgery involving colon resection and ileocolic anastomosis. The ileal loops in the hernia sac likely had a compromised venous drainage because of the volvulus. Following SMA stenting, the blood flow into the hernia sac increased, as the distal SMA and its branches opened up in the post-stenting angiogram. This led to venous congestion in the hernia as its venous drainage had already been compromised by the volvulus.

Reperfusion injury following stenting for CMI is rare. Abdominal pain and distension within 48 hours of mesenteric stenting have been reported as symptoms of reperfusion injury, and such patients recover following bowel rest and fluid resuscitation [4,6,7]. There are a few case reports of reperfusion hemorrhage occurring after mesenteric stenting. While two cases were managed conservatively, one was treated with endovascular embolization [5,8]. Reperfusion hemorrhage occurs due to increased mesenteric blood flow after stenting, just as venous congestion occurred in our case.

The imaging features on post-stenting CT observed in our case were similar to those of a reperfusion injury [3]. However, in our case, the patient developed symptoms two days after the procedure, unlike reperfusion injury, which usually presents within 48 hours of revascularization [3]. Further, dilatation of the mesenteric veins in the hernia sac was evident, suggesting venous congestion. The findings were also confirmed intra-operatively, and his symptoms resolved after the hernia repair.

Our case provides new insights into the management of CMI with concomitant internal hernia. Mesenteric revascularization can aggravate venous congestion within the hernia sac, especially in the case of a volvulus. Therefore, surgical repair of the internal hernia can be done before mesenteric revascularization to avoid such a complication.

## Conclusions

Endovascular stenting is the treatment of choice for CMI. Mesenteric revascularization increases the blood flow into hernia sacs, which may result in venous congestion in the bowel loops contained in the sacs. Early identification and treatment are essential in preventing strangulation and bowel ischemia. Such a complication needs to be kept in mind while treating CMI patients with associated internal hernias. Surgical repair of the internal hernia before mesenteric revascularization will help in avoiding it.
